# Changes in Typical Portion Sizes of Commonly Consumed Discretionary Foods among Australian Adults from 1995 to 2011–2012

**DOI:** 10.3390/nu9060577

**Published:** 2017-06-06

**Authors:** Miaobing Zheng, Anna Rangan, Beth Meertens, Jason H. Y. Wu

**Affiliations:** 1 The George Institute for Global Health, Faculty of Medicine, University of New South Wales, Sydney 2042, Australia; jwu1@georgeinstitute.org.au; 2 Charles Perkins Centre, School of Life and Environmental Sciences, University of Sydney, Sydney 2006, Australia; anna.rangan@sydney.edu.au; 3 National Heart Foundation of Australia, Brisbane 4006, Australia; beth.meertens@heartfoundation.org.au

**Keywords:** portion size, Australian adults, trends, energy-dense nutrient-poor foods

## Abstract

This study aimed to examine the changes in typical portion sizes of commonly consumed discretionary foods among Australian adults from 1995 to 2011–2012. Data of adults (age ≥19 years) from the 1995 Australian National Nutrition Survey and 2011–2012 National Nutrition and Physical Activity Survey were used. Typical portion sizes (median portion) of fourteen discretionary foods that contributed the most to energy intake were determined. Ten out of fourteen food categories demonstrated a significant change in kJ per typical portion from 1995 to 2011–2012 (*p* ≤ 0.001). kJ per typical portion increased for pizza, cake, sausage, cereal bar, processed meat, ice cream and wine, with pizza and cake demonstrating the largest increases (+570 kJ and +950 kJ in 2011–2012, respectively; both +66% above 1995). In contrast, kJ per typical portion of pastry, snack food and potato fries decreased by 10–40% over time, and did not change for biscuit, chocolate, sugar-sweetened beverage and beer. Similar changes were observed for grams per typical portion consumed. Temporal trends in typical portion sizes were similar according to age group, gender and socioeconomic status. The findings suggest that population-wide strategies that enable consumers to choose smaller portions of discretionary foods are needed to reduce the excess consumption of these products.

## 1. Introduction

There is growing scientific evidence that portion size (defined as the amount of foods and beverages consumed per eating occasion) is an important determinant of energy balance [1,2]. In experimental settings, offering larger portions of foods and beverages leads to increased intake, without compensatory reduction in intake at subsequent meals. Such effect has been observed both in children and adults, across gender, and in those with different body weight [3,4,5,6]. Continuous availability of larger portions can, therefore, result in increased daily energy intake and the increased likelihood of obesity [1,2]. 

Consumption of large portions of discretionary foods, such as sugary drinks and confectionery, has been highlighted as a particular cause for concern by recent research [2,7]. Currently, across many countries, the intake of discretionary foods and beverages is excessive [8,9]. Given the important role of portion size control for maintaining energy balance, there is a need to investigate the typical portion sizes of discretionary products, and how this may have changed over time. Identifying discretionary food categories with large typical portions, and those that have increased most over time, will help to inform the design and implementation of policies targeting portion sizes [10]. Only a handful of studies have assessed typical portion sizes of discretionary foods and beverages and their trends, using nationally representative surveys, with most studies based in the USA and the UK [11,12,13,14,15]. These investigations have found differences in both the magnitude and direction of change in typical portion sizes for specific discretionary foods between countries, highlighting the importance to conduct country-specific investigations. 

In Australia, the most recent nationally representative survey in 2011–2012 found that discretionary products contributed to about one-third of daily energy intake [16], despite dietary guidelines recommending to minimise the intake of such foods. The Australian Dietary Guidelines recommend that discretionary foods should be limited to small amounts and infrequently. The recommended ranges for male and female adults are 0–3 serves/day and 0–2.5 serves/day, respectively, depending on activity, height and weight range (one serve = 600 kJ) [17]. Importantly, there has been no longitudinal assessment of changes in the typical portion sizes among Australian adults. In order to address this gap in knowledge, the current study investigated trends in portion sizes of discretionary foods among adults from 1995 to 2011–2012 using Australian nationally representative surveys. To gain insight into whether such trends may differ by key demographic factors, we also assessed typical portion size trends according to age, gender, and socioeconomic status (SES).

## 2. Materials and Methods 

### 2.1. Survey Description

Data from two Australian nationally representative surveys: 1995 National Nutrition Survey (1995 NNS) and 2011–2012 National Nutrition and Physical Activity Survey (2011–2012 NNPAS) were used in the current analyses to track portion size trends over time. The 1995 NNS was conducted throughout Australia between February 1995 and March 1996 and used a structured three-pass, face-to-face 24-h recall to collect dietary intake. The 2011–2012 NNPAS was conducted from May 2011 to June 2012 and dietary intake was assessed using a five-phase automated multiple-pass 24-h face-to-face dietary recall supplemented with a second telephone 24-h recall. The 24-h recall was conducted by trained interviewers and was spread across all days of the week and four seasons. The distribution of the day and seasons of survey were similar between the two surveys [18]. The response rate for the 1995 NNS and 2011–2012 NNPAS was 61.4% and 77% respectively. A stratified multistage area sampling was used for sample selection in both surveys and to ensure the selected sample was representative of the Australian population. Participants were asked to report all foods and beverages consumed on the day prior to the interview, from midnight to midnight. As the 1995 NNS collected only one day dietary recall from most of the sample, the day one 2011–2012 NNPAS dietary intake data were used in the current analysis for comparison. The foods and beverages were translated into nutrient intakes using Australian Food, Supplement and Nutrient Database (AUSNUT) databases, and AUSNUT measurement files were used to convert household measurements to gram weight. The coding and data processing approach is largely consistent between the two surveys [18]. Detailed comparison of the two surveys pertaining to the study design and operation has been reported on the Australian Bureau of Statistics (ABS) website [18]. 

### 2.2. Assessment of Portion Size 

The 1995 NNS assessed portion sizes using standardized measuring guides including cups, spoons, ruler, measuring sticks, a grid, different-sized shapes and containers, and photos of selected food items. The 2011–2012 NNPAS utilised a more comprehensive food model booklet that contains a wider range of Australian-sourced food and beverage containers, mounds of various sizes, ruler guide, rings, a grid, a wedge, beef and chicken cuts and chocolate bar sizes to assist participants with portion size estimation [18]. Portion size is defined as the amount of a food that is consumed per eating occasion. In both surveys, respondents were asked for the time they began eating or drinking each food as well as what the respondent would call each eating occasion. If an individual consumed a food item on multiple occasions during the day, the average portion size for those multiple occasions was calculated and treated as a single record for that individual.

### 2.3. Definition and Classification of Discretionary Foods and Beverages

The 2013 Australian Dietary Guidelines [17] define discretionary foods as energy dense and nutrient poor foods and beverages that are not an essential part of a healthy dietary pattern. They are usually high in saturated fat, sugars, salt, and/or alcohol. Discretionary food codes were identified based on the ABS discretionary food list [19]. Fourteen discretionary food categories that were consumed by ≥5% of the total population and contributed most to the energy intake in 2011–2012 NNPAS were included in the current analyses [16]. These are pizza, cake, ice cream, sausage, processed meat, cereal bar, chocolate, biscuits, pastry, snack food, wine, beer and sugar-sweetened beverages (SSB). A list of foods that were included in each food category is presented in Table S1. Food categories between the two surveys were matched using the AUSNUT 2011–2012 and AUSNUT 1999 matching file at the eight-digit food code level to ensure comparable comparisons. Detailed food classification and categorisation methodology has been reported previously [20].

### 2.4. Statistical Analysis

Day one dietary data of adults aged 19 years and over from the 1995 NNS (*n* = 10851) and the 2011–2012 NNPAS (*n* = 9341) were used. Descriptive data for age, gender, SES, body mass index and macronutrient intakes for the 1995 and 2011–2012 survey participants were provided, and differences in means and proportions assessed using t-test and chi-squared test, respectively. Typical portion sizes were defined and calculated as median portion sizes in both kJ and grams, as the portion sizes of some food categories were not normally distributed. Personal weighting factors were applied to the data to ensure that the survey estimates conform to the population estimates by gender, age, area of usual residence and seasonal effects [18]. Percentage difference between typical portion sizes of 2011–2012 and 1995 was calculated as the difference between the 2011–2012 and 1995 typical portion sizes, divided by 1995 typical portion size, and multiplied by 100. Differences between the typical portion sizes over time were tested using the Mann–Whitney U test. To understand how changes in energy density may affect changes in typical portion sizes expressed in kJ, we also calculated the average energy density of key discretionary food categories. Energy densities for individual products within each food category were obtained from the 1999 and 2011–2012 AUSNUT food nutrient database.

Typical portion sizes were also examined separately by age groups (19 to 30 years, 31 to 50 years, 51 to 70 years, and ≥71 years) in accordance with the ABS Nutrient First Result report [16], gender (male and female), and SES (as defined by the Socio-Economic Indexes for Areas (SEIFA)) quintiles with the lowest quintile representing the most disadvantaged and highest quintile representing the most advantaged [18]. All statistical analyses were performed using SPSS 20.0 (SPSS Inc., Chicago, IL, USA) with the statistical significance set as *p* < 0.05 (two-sided). 

## 3. Results

### 3.1. Survey Participant Characteristics

Table 1 shows the survey participant characteristics in 1995 and 2011–2012. The proportion of males and females was not significantly different between two surveys. Participants were slightly younger (mean difference [95% CI], −1.82 [−2.31, −1.35] years) and had lower body mass index (mean difference [95% CI], −0.96 [−1.11, −0.82] kg/m^2^) in the 1995 NNS compared to the 2011–2012 NNPAS. The self-reported daily energy intake was lower in 2011–2012 compared to 1995 (mean difference [95% CI], −0.57 [−0.56, −0.67] MJ) and there were small differences in the percent of energy from macronutrients.

### 3.2. Overall Portion Size Trends

**Figure 1:**
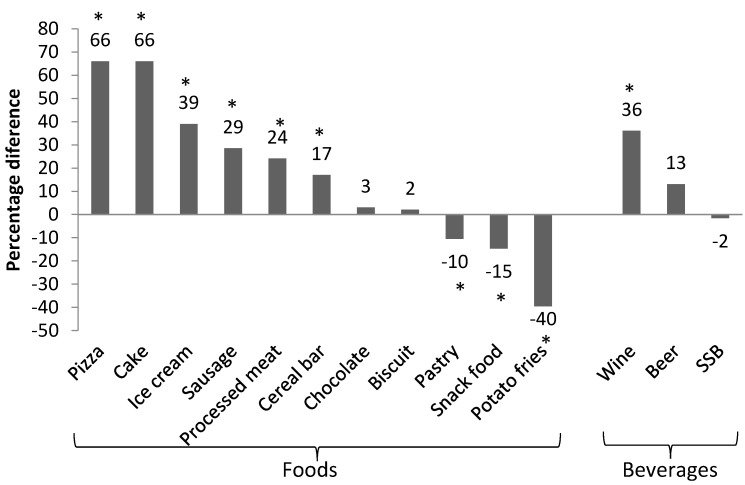
Percentage difference in median portion size (kJ) between 1995 and 2011–2012 by food category. * *p* < 0.05.

Percentage difference in the typical portion size (defined as the median portion size) of discretionary foods and beverages in kJ between 1995 and 2011–2012 is presented in Figure 1, and the actual values of the typical portion sizes are shown in Table S2. Seventy-one percent (10 out of 14) of the food categories exhibited significant changes in typical portion sizes between 1995 and 2011–2012. Typical portion sizes in kJ increased between 17% and 66% for pizza, cake, sausage, processed meat, ice cream, cereal bar, and wine (all *p* ≤ 0.001). Typical portion sizes in kJ decreased by 10–40% for pastry, snack food and potato fries. There was no appreciable change in typical portion size in kJ for chocolate, biscuit, beer and SSB. 

Generally, similar patterns of change were observed when typical portion sizes were expressed in grams rather than kJ (Table 2). Median portion sizes (in grams) of pizza, cake, sausage, processed meat, ice cream and wine were higher in 2011–2012 by 14–54% (all *p* ≤ 0.001). Conversely, median portion sizes in grams of pastry, snack food and potato fries were smaller in 2011–2012 by 13–39% (all *p* < 0.05). Notably, although typical portion sizes in kJ increased for cereal bar (Figure 1), when expressed as grams, there was no significant change (*p* > 0.05; see Table 2). Biscuit, chocolate, SSB, and beer also had similar typical portion sizes in grams over time. 

### 3.3. Trend in Energy Density of Discretionary Foods and Beverages

Table 3 presents the average energy density of key discretionary foods and beverages in 1995 and 2011–2012. Average energy density was similar for all product categories except for sausages and cereal bar. Energy density of sausage had decreased significantly from 1995 to 2011–2012 by a mean difference (95% CI) of −142 (−232, −52) kJ/100 g (*p* = 0.003). However, cereal bar had increased its energy density by a mean difference (95% CI) of 145 (43,247) kJ/100 g (*p* = 0.007).

### 3.4. Portion Size Trend by Sociodemographic Factors

**Figure 2:**
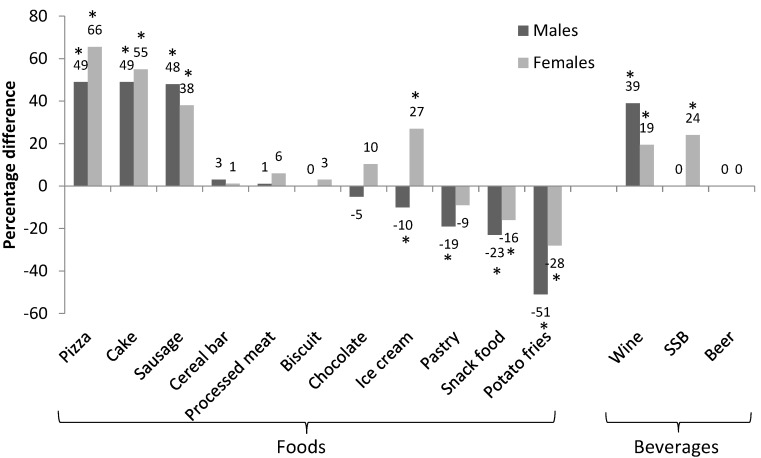
Percentage difference in median portion size (in grams) between 1995 and 2011–2012 by gender. * *p* < 0.05.

Percentage differences in median portion size in grams between 1995 and 2011–2012 by gender are illustrated in Figure 2. The majority of food categories (11 out of 14) revealed consistent change in portion size between genders. Both males and females increased their typical portion sizes for pizza, cake, sausage, and wine, decreased for snack food and potato fries, and maintained similar typical portion size for cereal bar, processed meat, biscuit, chocolate, and beer over time. Opposite typical portion size trends between genders were found for ice cream (male decreased by 10%, female increased by 27%), and SSB (male stable, female increased by 24%).

**Figure 3:**
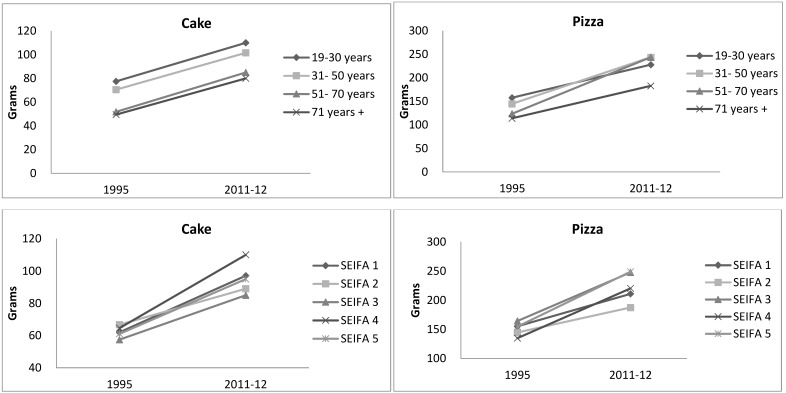
Typical portion size trends of cakes and pizza from 1995 to 2011–2012 according to age and Socio-Economic Indexes for Areas (SEIFA) groups. * *p* < 0.05.

Percentage differences between typical portion sizes in grams between 1995 and 2011–2012 by age and SEIFA groups are shown in Table S3. Changes in the typical portion size of most food categories were in the same direction among age and SEIFA groups such as pizza, cake, sausage, potato fries, wine and beer. For instance, typical portion size trends of pizza and cake were similar in direction and magnitude among age and SEIFA groups (Figure 3). Differential effects of age and SEIFA were noted for only a few discretionary food categories, which for the most part still demonstrated similar trends, but differed in their magnitude of change between 1995 and 2011–2012. For example, portion size of biscuit increased in the young adults (19–30 years), but remained similar for older age groups (31 years and over). 

## 4. Discussion

Using nationally representative data, the present investigation identified significant changes in typical portion sizes of discretionary foods over the last two decades for Australian adults. For half of the discretionary food categories examined, there were significant increases in the amount of energy per typical portion (pizza, cake, sausage, cereal bar, processed meat, ice cream and wine), although decreased energy per typical portion was also noted for three product categories (pastry, snack food and potato fries). Energy per typical portion was similar for four food categories (biscuit, chocolate, sugar-sweetened beverage and beer). Our findings suggest changes in energy per typical portion were likely to have been predominantly driven by the changes in the weight or volume of the discretionary products consumed per eating occasion, rather than changes in the energy density of such products themselves. Our results indicate that the changes in typical portion sizes were generally similar for Australians of different age groups, gender, and SES.

Increased typical portion sizes of several of the discretionary foods and beverages in Australia have important public health implications. Increases in the typical portion sizes of discretionary foods and beverages will not necessarily lead to increased total energy intake per eating occasion or per day [13], as other factors may influence energy intake, such as frequency of consumption and the overall composition of a meal. Nevertheless, portion size is a key variable (per eating occasion and over the day) with experimental studies consistently suggesting larger portion sizes can override self-regulation of energy balance and contribute to increased total energy intake and weight gain [1,2]. Analysis of longitudinal surveys in the United States also supports the likelihood that an increase in typical portion sizes is a key factor contributing to increased energy intake over the past several decades [21]. As discretionary foods are usually also high in saturated fats, added sugars, and salt, increased typical portion sizes could also contribute to excess intakes of these nutrients and increase cardio-metabolic disease risk. Additionally, health guidelines generally recommend consuming no more than two standard drinks per day (e.g., 2 × 100 mL wine, or 2 × 375 mL mid-strength beers) for adults to reduce the risk of alcohol related harm [22]. In relation to these recommendations, the large typical portion sizes of beer and wine in 1995, and the observed additional increase in typical portion size for wine between 1995 to 2011–2012 for Australian adults, are also concerning.

A reduction in typical portion sizes of pastry, snack food and potato fries from 1995 to 2011–2012 among Australian adults was a novel finding. The large magnitude (−40%) of decrease for a typical portion size of potato fries was particularly notable. Aligned with our findings, Collins et al reported that among Australian children aged 2–16 years, there was an increase in portion sizes of pizza, a decrease in portion sizes of snack food and potato fries, and no change in chocolate and SSB typical portion sizes, from 1995 to 2007 [23]. It is possible that increased awareness or perception by the public of pastries, snack foods, and potato fries as unhealthy foods may have contributed to changing preferences towards smaller portion sizes over time, or increased under-reporting of portion sizes for these products. 

Congruent with our results, prior studies found that trends in typical portion sizes in adults over time were generally consistent across age, gender, and SES groups [11,12]. In contrast, prior research suggests that older adults and women generally have better dietary quality than younger adults and men, and temporal trends in dietary quality for groups from different SES have widened over time [24,25,26]. Similar trends in typical portion sizes across distinct sub-populations suggest that consumer portion size selection may be strongly influenced by environmental factors such as availability of large portion sizes of meals and packaged foods, and price discounting of larger portion size products [27].

With the growing prevalence of obesity and chronic diseases, local and national governments in many countries are investigating policies to reduce portion sizes of discretionary foods and beverages [10]. Recent examples include the City of New York’s attempted portion size limits on sugary drinks, and the portion size working group convened by the Australian government in 2016 as part of its Healthy Food Partnership initiative [28]. The substantial increases in typical portion sizes of several discretionary foods and beverages observed in this investigation, supports the need for stakeholders to develop strategies to counter such trends. Our findings, that increases in typical portion sizes of discretionary foods are frequently consumed away from home (e.g., pizza, cake and ice cream), suggest that food service providers could be encouraged to adopt marketing and operational strategies to help consumers with portion size control [29], such as offering smaller portion sizes of take-away foods and fast food items as the default (i.e., “downsizing”). The relatively stable typical portion sizes for products commonly obtained from supermarkets and other retail outlets (biscuit, chocolate, and SSB) suggest consumer portion size selection could be significantly determined by the package size available—and highlight an opportunity to engage with food manufacturers to provide and promote smaller packaged products in these product categories. Some companies in Australia and overseas have voluntarily pledged to reduce package size in various discretionary foods [10]. However, the effectiveness of such an approach remains unclear due to the lack of clearly defined portion size targets and monitoring/reporting mechanisms [10]. The role of labelling continues to be investigated, with recent policy developments in Canada and United States proposing that serving size information be consistent with what people typically eat (e.g., two slices of bread instead of one) [30,31,32], although the efficacy for such an approach to encourage appropriate portion size selection also remains unclear. Increasingly, healthy food and drink criteria are emphasising limits on portion sizes for discretionary products allowed in the settings of schools, health facilities and workplaces [33]. Implementation of such policies may be especially feasible in public sector institutions, which could help change the accepted portion size norms and increase incentive for the food industry to develop smaller packs of discretionary products [27]. It is unlikely that one strategy will successfully address the disparity between discretionary foods and beverages contributing approximately one-third of daily energy and recommendations to minimise intake of these foods. Changes to portion size should be progressed alongside other strategies, including reformulation, labelling and restrictions on marketing of unhealthy food to children [34]. A multi-component, population-based strategy that engages with the food industry will likely be required [35].

This study has several strengths. The assessment of portion sizes in the nutrition surveys used validated instruments which reduces measurement error [18]. The large sample sizes of the survey datasets increase the precision of the estimation of population typical portion sizes. The sampling methodology of the nationally representative survey ensures these findings are generalisable to the Australian population. 

Limitations of this study should also be noted. Portion sizes were self-reported rather than objectively measured, which introduces recall and measurement error. The two dietary surveys used were similar, but not identical, in terms of methods used to assess dietary intake and portion size, which could have affected the results—for example, in 2011, a more comprehensive food model booklet with a wider set of food and beverage containers and food images were used to assist with portion size estimations. Our analyses were based on a single 24-h recall and does not infer usual intake, but this method is considered as a valid measure for estimating mean intake at the group level [18]. Underreporting of discretionary foods and beverages in a socially desirable way is highly likely [36]. However, the extent to which people misreport portion size is hard to estimate, and this would be a problem for both surveys. Location of consumption was not recorded in both dietary surveys, so it was not possible to examine whether typical portion sizes differed by setting (e.g., home or eating out), and this should be investigated in future studies.

## 5. Conclusions

In conclusion, typical portion sizes have increased for a large proportion of commonly consumed discretionary foods and beverages for Australian adults from 1995 to 2011–2012. The generally consistent patterns of change in typical portion sizes across age, gender, and groups of varying socioeconomic status, suggest that population-wide strategies that enable consumers to choose smaller portions of discretionary foods and drinks are needed to reduce the excess level of consumption of these products. These findings highlight the need for ongoing, high quality dietary surveys over time to monitor future trends in typical portion sizes and evaluate the efficacy of policy interventions.

## Figures and Tables

**Table 1:** Survey participant characteristics in 1995 and 2011–2012.

-	1995 NNS	2011–2012 NNPAS	*p*-Value
Gender (%)			
Male	46.8	45.8	0.165
Female	53.2	54.2	
Age (years)	44.5 (17.2)	46.3 (17.4)	<0.0001
SEIFA quintile (%)			
1	18.3	18.8	<0.0001
2	19.6	20.8	
3	19.3	19.8	
4	21.2	17.7	
5	21.6	22.8	
Body mass index (kg/m^2^)	26.3 (4.7)	27.3 (5.5)	<0.0001
Energy intake (MJ/day)	9.2 (4.1)	8.7 (3.7)	<0.0001
Protein (%E)	17.1 (4.8)	18.4 (6.2)	<0.0001
Fat (%E)	32.5 (8.4)	30.8 (8.9)	<0.0001
Carbohydrate (%E)	46.0 (9.9)	43.5 (11.0)	<0.0001

1995 NNS: National Nutrition Survey; 2011–2012 NNPAS: National Nutrition and Physical Activity Survey; SEIFA: Socio-Economic Indexes for Areas.

**Table 2:** Comparison of typical portion sizes (in grams) of discretionary foods between 1995 and 2011–2012.

Food Categories	1995					2011					Percentage Difference between Median
	*n*	Median (g)	IQR	Mean (g)	SD	*n*	Median (g)	IQR	Mean (g)	SD	
Foods											
Pizza	485	148	91, 260	194	154	466	228	144, 328	255	151	54 *
Cake	2467	62	36, 99	80	69	1540	95	56, 143	108	73	53 *
Sausage	974	90	57, 141	108	81	611	114	89, 178	145	80	27 *
Processed meat	3280	29	18, 48	39	39	1989	34	17, 52	45	42	17 *
Ice cream	1436	73	46, 124	96	75	1149	83	66, 132	104	71	14 *
Cereal bar	181	31	31, 32	35	13	483	32	31, 42	40	17	3
Biscuit	3960	20	13, 33	26	21	3150	20	14, 34	28	28	0
Chocolate	2070	24	13, 45	34	33	2138	24	13, 45	35	38	0
Pastry	1840	150	89, 186	158	110	1113	130	67, 175	138	100	−13 *
Snack food	796	30	21, 50	40	33	827	25	17, 50	44	47	−17 *
Potato fries	1567	120	100, 150	133	75	1045	73	46, 122	99	90	−39 *
Beverages											
Wine	1549	249	166, 375	307	227	1555	297	208, 488	390	290	19 *
SSB	3338	350	261, 469	401	267	2763	374	270, 468	424	269	7
Beer	1804	755	378, 1134	941	826	1249	758	379, 1137	955	840	0

IQR: interquartile range, SD standard deviation; * *p* < 0.05.

**Table 3:** Comparison of average energy density (energy per 100 g) of key discretionary food categories that demonstrated ≥15% difference in typical portion size (kJ) between 1995 and 2011–2012.

	1995			2011–2012			Mean Difference (95% CI)
	*n*	Mean (kJ)	SD	*n*	Mean (kJ)	SD	
Pizza	25	1076	120	63	1073	110	−3 (−55, 50)
Cake	238	1519	306	188	1502	215	−17 (−69, 34)
Sausage	29	1120	120	30	978	211	−142 (−232, −52) *
Ice cream	69	865	257	63	846	211	−19 (−100, 63)
Cereal bar	8	1063	97	43	1748	237	145 (43, 247) *
Potato fries	46	1059	314	35	1023	244	−36 (−164, 91)
Wine	10	254	48	12	294	61	40 (−10, 90)

Energy densities for individual products within each food category are obtained from the 1999 and 2011–2012 AUSNUT food nutrient database. *n*: number of food items. SD standard deviation, IQR: interquartile range. * *p* < 0.05.

## Supplementary Materials

The following are available online at www.mdpi.com/2072-6643/9/6/577/s1, Table S1: Discretionary food categories included in this analysis, Table S2: Comparison of typical portion sizes (in kJ) of discretionary foods in 1995 and 2011–2012, Table S3: Percentage difference in typical portion size (g) between 1995 and 2011–2012 by age and socioeconomic status groups.
