# Supplementary material for: Changes in Typical Portion Sizes of Commonly Consumed Discretionary Foods among Australian Adults from 1995 to 2011–2012
Source: Nutrients. 2017 Jun 6;9(6):577. doi: 10.3390/nu9060577 (PMC5490556; doi:10.3390/nu9060577)
Supplement: Supplementary file 1 [file nutrients-09-00577-s001.zip › nutrients-195652-supplementary.pdf]

Supplementary materials

Table S1. Discretionary food categories included in this analysis

**Discretionary food categories**	**Subcategories**
Pizza	Thin or thick crust, any topping
Cake	Cakes, cake-type muffins, slices, scones and rock cakes
Sausage	Sausages, frankfurts and saveloys
Processed meat	Bacon, ham and luncheon meats
Ice cream	Vanilla ice cream, chocolate ice cream, other sweet-based ice cream, fruit-flavoured ice cream, ice cream cakes/cups/ cones/stick, gelato
Cereal bar	Muesli and cereal bar
Biscuit	Sweet biscuits: chocolate, jam filled, fruit filled, choc-chip, plain sweet unfilled, chocolate coated, Shortbread style, Gingerbread, fruit and nuts, other toppings; Savoury biscuits: plain energy>1800kJ/100g
Chocolate	Chocolate-based confectionery: plain chocolate, chocolate with nuts, chocolate bars, other chocolate-based confectionery
Pastries	Sweet pastries, fruit or nut filling, egg or dairy based filling, quiches and flans, savoury pastries, pies, rolls and envelopes
Snack food	Potato crisps, popcorn, corn chips, extruded snacks
Potato fries	Fries and wedges
Wine	Red wine, rose, white wine and sparkling
Sugar-sweetened beverage	Soft drinks, fruit drinks, cordials, sport/energy drinks
Beer	Regular and reduced alcohol beer

Table S2. Comparison of typical portion sizes (in kJ) of discretionary foods in 1995 and 2011-12

Food categories	1995					2011					Percentage difference between median
	n	**Median (kJ)**	IQR	**Mean (kJ)**	SD	n	**Median (kJ)**	IQR	**Mean (kJ)**	SD	
Foods											
Pizza	485	**1432**	883 , 2539	**1919**	1519	466	**2382**	1559 , 3613	**2757**	1651	**66***
Cake	2467	**868**	515 , 1431	**1143**	1002	1540	**1437**	798 , 2136	**1584**	1088	**66***
Ice cream	1436	**575**	342 , 980	**772**	609	1149	**802**	450 , 1040	**852**	554	**39***
Sausage	974	**945**	591 , 1418	**1153**	856	611	**1215**	913 , 1994	**1530**	863	**29***
Processed meat	3280	**188**	104 , 352	**299**	391	1989	**234**	79 , 479	**374**	446	**24***
Cereal bar	181	**481**	465 , 541	**554**	195	483	**562**	531 , 771	**689**	307	**17***
Chocolate	2070	**428**	233 , 870	**627**	633	2138	**441**	239 , 882	**663**	720	**3**
Biscuit	3960	**380**	255 , 625	**501**	425	3150	**388**	252 , 685	**533**	547	**2**
Pastry	1840	**1599**	1035 , 1956	**1674**	1125	1113	**1431**	943 , 1650	**1420**	871	**-10***
Snack food	796	**630**	447 , 1085	**1479**	826	827	**538**	346 , 1058	**1040**	910	**-15***
Potato fries	1567	**1440**	1030 , 1746	**857**	693	1045	**870**	449 , 1393	**913**	953	**-40***
Wine	1549	**707**	471 , 1062	**856**	634	1555	**962**	642 , 1534	**1209**	901	**36***
Beer	1804	**890**	563 , 1690	**1308**	1183	1249	**1007**	542 , 1625	**1308**	1170	**13**
SSB	3338	**598**	427 , 785	**668**	448	2763	**589**	437 , 718	**648**	413	**-2**

IQR: interquartile range, SD standard deviation

*P<0.05

Table S3. Percentage difference in typical portion size (g) between 1995 and 2011-2012 by age and socioeconomic status groups

	Age groups				Socioeconomic status (SES)				
	19-30y	31-50y	51-70y	71y+	SEIFA 1	SEIFA 2	SEIFA 3	SEIFA 4	SEIFA 5
Foods									
Pizza	45*	68*	97*	60*	36*	30*	51*	63*	61*
Cake	42*	44*	64*	62*	57*	33*	48*	70*	56*
Sausage	62*	50*	25*	20*	7*	15*	20*	69*	31*
Processed meat	7	17*	20*	10	10	13	11	13	25*
Ice cream	-11	13	33*	20*	25*	8	-5	18*	21*
Cereal bar	3	1	10	0	-3	3	10	5	3
Biscuit	20*	-5	4	12	4	0	9	-5	-6
Chocolate	-6	0	15	33*	0	9	9	-6	0
Pastry	-19*	-15*	-2	-19*	1	-21*	-9	-29*	-19*
Snack food	-11	0	-14	-18	-10	-7	-36*	-3	-17*
Potato fries	-34*	-35*	-38*	-43*	-39*	-35*	-39*	-38*	-41*
Beverages									
Wine	20*	20*	25*	31*	3	29*	19*	20*	19*
SSB	0	15*	38*	0	-7	4	12	-1	19*
Beer	-12	0	0	6	0	1	0	-11	-13

*P<0.05 Comparison of typical portion size from 1995 to 2011-12 among age and SES groups
